# Leveraging explainable artificial intelligence to optimize clinical decision support

**DOI:** 10.1093/jamia/ocae019

**Published:** 2024-02-22

**Authors:** Siru Liu, Allison B McCoy, Josh F Peterson, Thomas A Lasko, Dean F Sittig, Scott D Nelson, Jennifer Andrews, Lorraine Patterson, Cheryl M Cobb, David Mulherin, Colleen T Morton, Adam Wright

**Affiliations:** Department of Biomedical Informatics, Vanderbilt University Medical Center, Nashville, TN 37203, United States; Department of Computer Science, Vanderbilt University, Nashville, TN 37212, United States; Department of Biomedical Informatics, Vanderbilt University Medical Center, Nashville, TN 37203, United States; Department of Biomedical Informatics, Vanderbilt University Medical Center, Nashville, TN 37203, United States; Department of Medicine, Vanderbilt University Medical Center, Nashville, TN 37203, United States; Department of Biomedical Informatics, Vanderbilt University Medical Center, Nashville, TN 37203, United States; Department of Computer Science, Vanderbilt University, Nashville, TN 37212, United States; School of Biomedical Informatics, University of Texas Health Science Center, Houston, TX 77030, United States; Department of Biomedical Informatics, Vanderbilt University Medical Center, Nashville, TN 37203, United States; Department of Pediatrics, Vanderbilt University Medical Center, Nashville, TN 37203, United States; Department of Pathology, Microbiology and Immunology, Vanderbilt University Medical Center, Nashville, TN 37203, United States; HeathIT, Vanderbilt University Medical Center, Nashville, TN 37203, United States; Department of Psychiatry and Behavioral Sciences, Vanderbilt University Medical Center, Nashville, TN 37203, United States; HeathIT, Vanderbilt University Medical Center, Nashville, TN 37203, United States; Department of Medicine, Vanderbilt University Medical Center, Nashville, TN 37203, United States; Department of Biomedical Informatics, Vanderbilt University Medical Center, Nashville, TN 37203, United States; Department of Medicine, Vanderbilt University Medical Center, Nashville, TN 37203, United States

**Keywords:** clinical decision support, explainable artificial intelligence, electronic health record

## Abstract

**Objective:**

To develop and evaluate a data-driven process to generate suggestions for improving alert criteria using explainable artificial intelligence (XAI) approaches.

**Methods:**

We extracted data on alerts generated from January 1, 2019 to December 31, 2020, at Vanderbilt University Medical Center. We developed machine learning models to predict user responses to alerts. We applied XAI techniques to generate global explanations and local explanations. We evaluated the generated suggestions by comparing with alert’s historical change logs and stakeholder interviews. Suggestions that either matched (or partially matched) changes already made to the alert or were considered clinically correct were classified as helpful.

**Results:**

The final dataset included 2 991 823 firings with 2689 features. Among the 5 machine learning models, the LightGBM model achieved the highest Area under the ROC Curve: 0.919 [0.918, 0.920]. We identified 96 helpful suggestions. A total of 278 807 firings (9.3%) could have been eliminated. Some of the suggestions also revealed workflow and education issues.

**Conclusion:**

We developed a data-driven process to generate suggestions for improving alert criteria using XAI techniques. Our approach could identify improvements regarding clinical decision support (CDS) that might be overlooked or delayed in manual reviews. It also unveils a secondary purpose for the XAI: to improve quality by discovering scenarios where CDS alerts are not accepted due to workflow, education, or staffing issues.

## Introduction

The federal government has spent more than $34 billion on the implementation of electronic health records (EHRs) over the past decade.^1^ Clinical decision support (CDS) alerts, a critical component of EHRs, provide patient-specific information paired with organized clinical knowledge to reduce errors and improve healthcare quality.^2^ However, most alerts are infrequently accepted (acceptance rates < 10%), which leads to alert fatigue and desensitizes clinicians to alerts of higher importance.^3–6^ Additionally, many alerts fire at inopportune times (eg, a weight-loss alert during a cardiac resuscitation) or in clinical scenarios where they are unlikely to be helpful (eg, a cholesterol screening alert for a hospice patient).^7^ CDS alerts are often triggered by a limited set of criteria (eg, suggest cholesterol screening in men >35 or women >45 who have not received screening). Accounting for additional criteria (eg, exclude hospice patients from cholesterol screenings) could eliminate some alert firings that are extremely unlikely to be accepted.

Researchers have attempted various approaches to identify these criteria and improve alert quality, including manual review^8–11^ and collecting feedback from healthcare providers^12^^,^^13^ to adjust or turn off low-response alerts. These approaches are time- and labor-intensive, which prohibits rapid improvement of alerts. Additionally, manual reviews can only consider a small number of variables at a time, making it difficult to fully understand complex clinical scenarios. Finally, clinician feedback often fails to comprehensively capture all users’ perceptions, and it introduces recall bias.^14^ Previous research has found that clinician type, work complexity, and repeated alerts influence the perceived values of alerts.^15–17^ Combining alert log data with EHR data can provide information on who overrides alerts, when, and under what circumstances, which can be used to better target alerts.^18^ Therefore, an urgent need arises for an efficient and fair approach to comprehensively analyze user interaction with alerts and automatically generate suggestions to target alerts more precisely or improve clinical processes.

Explainable artificial intelligence (XAI) approaches are promising tools to address this need. XAI is a range of techniques designed to maintain high learning performance in AI while enabling users to understand model behavior.^19^^,^^20^ XAI techniques can be broadly categorized into 2 types based on the scope of their explanations: global and local.^21^ Global explanations focus on the entire model’s rationale, providing a comprehensive overview of the decision-making process and its various potential results. This kind of transparency is reflected in models such as logistic regression, where the entire logic must be clear and traceable. However, the pursuit of global explanations often leads to a trade-off with model complexity and predictive power. On the other hand, local explanations focus on explaining individual decisions or predictions. XAI techniques in this category tailor explanations to specific instances, offering justifications for the model’s behavior in particular scenarios. A prominent example of this is the Local Interpretable Model-Agnostic Explanations (LIME) technique, which provides local approximations of a model’s predictions, enabling a granular understanding of its operations.^22^ Building on such foundational work, new methods like “Anchors” promise to enhance the precision of these local explanations with decision rules, guiding users through the AI’s reasoning for individual cases.^23^

In prior work, we developed a traditional machine learning model that could suppress 294 871 (54.1%) medication alert firings while maintaining a false-negative rate of only 0.9% (ie, missed 430 acceptances) in a test dataset, illustrating that machine learning models based on the alert log data can accurately predict user response to alerts within a single organization.^24^ However, results from machine learning are insufficient in that they lack transparency and are difficult to integrate into current rule-based alerts. Using XAI techniques allows for explanations of models (ie, user acceptance or not-acceptance of alerts). For example, one potential explanation of the model is that the model’s prediction is to not accept the alert when the patient is in postpartum department for the Contraindicated—Non-steroidal anti-inflammatory drugs (NSAIDs) and Pregnancy alert. Based on this explanation, CDS experts could review the alert logic, for instance, by considering the patient’s presence in the postpartum department as an exclusion criterion for the Contraindicated—NSAIDs and Pregnancy alert to improve specificity of alerts and reduce unnecessary alerts. The purpose of this study was to develop and evaluate a data-driven process that generates suggestions to improve alert criteria.

## Materials and methods

### Method overview

**Figure 1.**
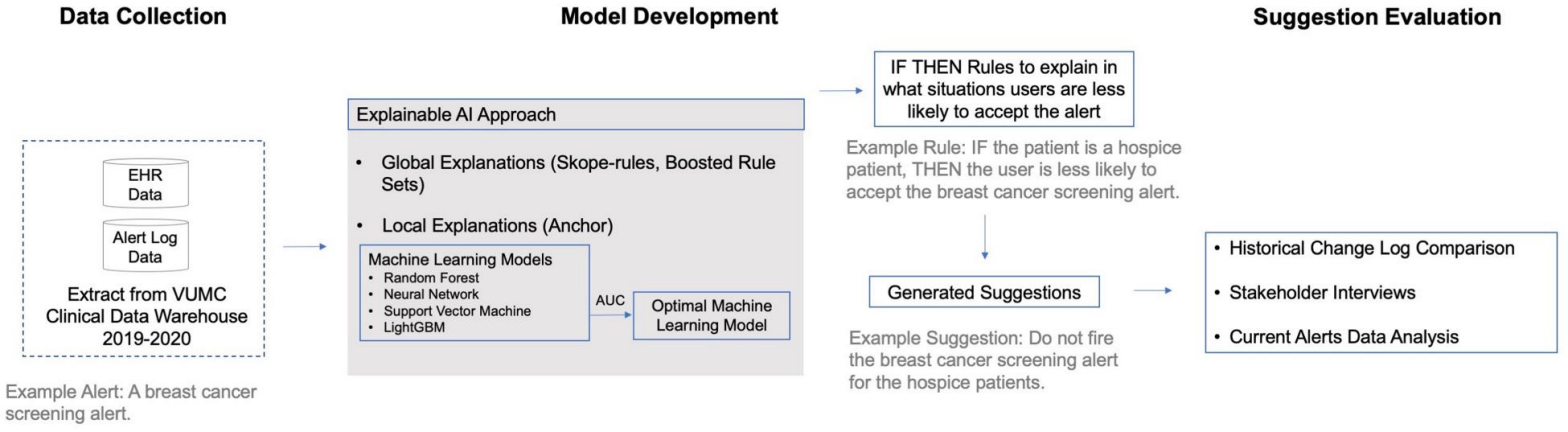
Study overview.

The method overview is shown in Figure 1. It consists of 3 components: (1) a data collection step that extracts alert log data and associated variables from EHR; (2) a model development component that applies XAI approaches to generate suggestions on improving the alert logic; and (3) a suggestion evaluation component, which includes: historical change log comparison, stakeholder interviews, and current alerts data analysis. For machine learning models used in the XAI approach, we used the Area under the ROC Curve (AUC) to select the optimal models. The output of models was a set of IF-THEN rules to explain in what situations users were less likely to accept the alert. We used a set of metrics—odds ratio (OR), probability of low acceptance, decrease rate, confidence, interest, conviction, and the *P* value of $\chi^{2}$—to select rules into the evaluation step. We then converted the rules into suggestions. For example, for a breast cancer screening alert, a rule was: IF the patient was a hospice patient, THEN the user was less likely to accept the alert. The corresponding suggestion would be: Do not fire the breast screening alert for hospice patients.

### Data collection

Leveraging a previously developed taxonomy to identify features influencing user responses to alerts, we extracted data from Vanderbilt University Medical Center (VUMC)’s Epic Clarity clinical data warehouse for alerts generated from January 1, 2019 to December 31, 2020. To build a predictive model, the dataset only included data generated before the alert was displayed to the user.^24^ Alerts with firing counts ≤100, or acceptance counts ≤10, were excluded. User responses were categorized as accepted or not accepted. If the user chose “Acknowledge/override Warning,” “Cancel Warning,” “No Action Taken,” “Accept BPA (No Action Taken),” or “Cancel BPA,” then the user response was classified as “not accepted.” Otherwise, the user response belonged to “accepted,” (ie, single order, remove order set). For the NSAIDs alert, for example, we extracted 2196 instances of the alert firing. Each row contained one alert firing and the outcome (in this case, if the NSAID was removed the alert was classified as “accepted”; if the user overrode the alert and kept the NAID order, it was counted as “not accepted”). For each alert firing record, we also extracted 2689 features, such as the patient’s age, all of their diagnoses, lab results, etc. The same features are used for every CDS alert. Numeric variables were binned into 10 groups with equal-width intervals.^25^

### Model development

Four machine learning techniques were evaluated: random forest, neural network, support vector machine, and gradient-boosted trees. These models are commonly used for medical data, and gradient-boosted tree models (LightGBM implementation) have demonstrated superior performance in recent studies.^24^^,^^26^^,^^27^ To impute missing values of numerical features, we compared mean imputation, median imputation, and imputation with the most frequent value. For categorical features, we used the one-hot encoding method.^28^

We applied 3 XAI techniques: skope-rules, boosted rule set, and Anchor. The skope-rules technique extracts rules from gradient-boosted trees, deduplicates them, and combines them based on out-of-bag precision, providing a global explanation that represents typical patterns across the entire dataset.^29^ On the other hand, the boosted rule set, employing the principles of AdaBoost, sequentially fits a set of rules to handle data variance effectively.^30^ Unlike gradient boosted trees, which focus on reducing residuals and typically produce deeper trees, AdaBoost emphasizes correctly classifying previously misclassified instances by adjusting the weights of observations and classifiers. Lastly, the Anchor technique is utilized for its ability to generate precise local explanations, which represent local patterns.^23^ We used optuna, InterpretML, and imodels packages in Python 3.10 to develop models.^31–33^

We merged the rules generated by the XAI approaches and removed duplicates. For each rule, we used a set of selection metrics to compute the values to select candidates for the final evaluation: odds ratio (OR), probability of low acceptance, decrease rate, confidence, interest, conviction, and the *P* value of $\chi^{2}$. The probability of low acceptance uses the Binomial distribution with a Beta(1,1) (ie, uniform) prior to identify alerts whose acceptance rate may fall below a certain threshold. To illustrate, consider the rules for improving alerts, such as “IF the patient is a hospice patient, THEN the user is less likely to accept the breast cancer screening alert.” Assuming the breast cancer screening alerts were triggered in 1000 hospice patients and accepted 16 times, the excluded firings count would be 1000, leading to a subpopulation acceptance rate of 1.6%. In this scenario, the posterior distribution of the acceptance rate would be Beta(17,985), giving a probability of 0.782 that the acceptance rate for these excluded firings would be at most 0.02, thereby supporting the adoption of the corresponding suggestion “Do not fire the breast cancer screening alert for the hospice patients.” This metric effectively accounts for both the number of firings (the more the better) and the number of acceptances (the fewer the better) when selecting obsolete alerts. Additional metrics were calculated as detailed in Table 1. The pros and cons of each metric are displayed in Table S1. We recognized the necessity of employing multiple metrics to evaluate the usefulness of suggestions in CDS alert optimization. Simple metrics were considered initially; however, they proved insufficient for a holistic assessment. Finally, a subset of 1000 randomly chosen suggestions was reviewed by 2 CDS experts (A.W. and A.B.M.), who examined each rule alongside its corresponding metric values, to determine the thresholds for both the beta probability distribution and the selection metrics. The thresholds were thus informed by the manual review, leveraging the expertise of our CDS experts to optimize for practical impact on CDS alerts improvement.

**Table 1.** Metric to select generated suggestions.

	X (Firings in the generated suggestion)	$\bar{X}\;$ (Firings not in the generated suggestion)
Y (not accepted)	*a*	*c*
$\bar{Y}$ (accepted)	*b*	*d*
	Odds ratio = $\frac{a\;*\;d}{b\;*\;c}$ Probability of acceptance Beta(1 + b, 1 + a) Decrease rate =$\frac{\left(a\;+\;b\right)}{\left(a\;+\;b\;+\;c\;+\;d\right)}$ Confidence = $\frac{a}{a\;+\;b}$ Interest = $\frac{\left(\frac{a}{a\;+\;b}\right)}{\left(\frac{a\;+\;c}{a\;+\;b\;+\;c\;+\;d}\right)}$ Conviction = $\frac{\left(a\;+\;b\right)\cdot (b\;+d\;)}{(a\;+\;b\;+\;c\;+\;d)\cdot b}$ *P* value of $\chi^{2}$	

### Suggestion evaluation

We conducted a comprehensive qualitative and quantitative evaluation of the generated suggestions. The first method we used in the qualitative assessment was to compare the generated suggestions with BPA’s historical change logs to identify consistent or partially consistent modifications and record the corresponding modification dates. The change log is a locally developed tool containing all changes made to BPAs in VUMC’s Epic system since 2019, generated each day by comparing the current version of each BPA record with the previous day’s version and recording the differences. Generated suggestions that matched or partially matched with BPA changes were classified as “helpful suggestions.” For the remaining generated suggestions, we conducted stakeholder interviews aimed at assessing clinical significance through the insights of relevant healthcare professionals. The generated suggestions that did not match BPA changes but were clinically correct were also classified as “helpful suggestions.” Notably, a limitation of the above methods was that generated suggestions with seemingly disparate modifications may have similar implications, posing a challenge to manual side-by-side comparisons. For example, a generated suggestion “exclude patients with chief complaints of ‘Return’ for an alert about Retinopathy of Prematurity (ROP) exam for premature infants” had the same effect as a modification to exclude all outpatients. Because in the clinical settings for this alert, the chief complaint “Return” was only used for outpatients. As a complementary approach, we extracted the most recent user response and corresponding features for alerts generated from March 1, 2023 to June 30, 2023, to assess whether alerts still fired in the context of the generated suggestions. In addition, for each BPA, we calculated the relative change in acceptance rate compared to the original acceptance rate.

## Results

The final dataset included 2 991 823 firings with 2689 features, 139 BPAs, 247 648 patients, and 18 397 users. The features are listed in Table S2. The overall acceptance rate was 12.3%. Among the machine learning models, the LightGBM model achieved the highest value in AUC: 0.919 [0.918, 0.920]. It was selected as the optimal machine learning model for generating suggestions in Anchor. Sensitivity, precision, F1, accuracy, and AUC for each machine learning model are listed in Table 2.

**Table 2.** Prediction results on the testing dataset.

Model	Sensitivity	Precision	F1	Accuracy	AUC
LightGBM	0.441 [0.437, 0.444]	0.737 [0.733, 0.741]	0.551 [0.548, 0.555]	0.912 [0.911, 0.912]	0.919 [0.918, 0.920]
Neural network	0.39 [0.387, 0.393]	0.681 [0.676, 0.686]	0.496 [0.493, 0.499]	0.902 [0.902, 0.903]	0.877 [0.876, 0.878]
Support vector machine	0.098 [0.095, 0.100]	0.612 [0.602, 0.620]	0.169 [0.165, 0.172]	0.881 [0.881, 0.882]	0.811 [0.809, 0.812]
Random forest	0.646 [0.643, 0.650]	0.55 [0.547, 0.553]	0.594 [0.591, 0.597]	0.891 [0.891, 0.892]	0.904 [0.903, 0.905]

Applying pre-defined thresholds (odd ratio > 1.25, probability of low acceptance > 0.5, decrease rate < 0.4, confidence > 0.98, interest > 1, conviction > 1.2, and *P*[$\chi^{2}$] < 0.01) and taking the intersection, a total of 1727 generated suggestions were selected as candidates for evaluation. In historical change log comparisons, we found that 76 of the suggestions either fully or partially matched with historical changes (63 fully matched and 13 partially matched). In addition, another 20 suggestions were identified as “helpful suggestions” in stakeholder interviews. Taken together, these 96 helpful suggestions were associated with 18 BPAs. Among 2 991 823 firings, 278 807 firings (9.3%) could have been eliminated. Table 3 shows examples of generated suggestions and their corresponding comments. All modified, partially modified, or discussed generated suggestions are listed in Table S3.

**Table 3.** Examples of generated suggestions and feedback from clinicians.

BPA	Generated suggestion	Comment
This patient has one or more Shared Plan of Care FYI flags which may require your attention. [High Priority]	Do not fire when: Encounter Type = Documentation	Already changed, the same exclusion was added on March 16, 2023.
This patient is due for the flu vaccine. Please order or specify why the vaccine cannot be ordered. [High Priority]	Do not fire when: Patient Department = VPH ADULT PARTIAL HOSPITALIZATION	Already changed, the same exclusion was added on December 17, 2020.
Contraindicated—NSAIDs and Pregnancy [Important]	Do not fire when: Patient Department = VUH 4E POST PARTUM	Already changed, add exclusion criteria: exclude Department = VUH 4E POST PARTUM on August 6, 2020.
Admission medication reconciliation is incomplete, there are home medications that need a decision. [High Priority]	Do not fire when: Provider Type = Nurse Anesthetist	Partial effect, the current BPA limits to Nurse Anesthetist with encounter type: hospital encounter
Warfarin Dosing Advisor [Critical] (an alert which recommends use of pharmacogenomic data to calculate initial warfarin dose)	Do not fire when: Encounter Type = Anticoagulation Visit	Discussed with clinicians, correct suggestion. “Almost none of those patients are new to warfarin therapy and so the anticoagulation clinic is going to use INR history for most patients.”
This patient is at risk for unintentional opioid overdose due to the following and a naloxone prescription is required to be offered by TN law. [Important]	Do not fire when: Encounter Type = Refill	Discussed several times at the CDS committee meeting, where opinions were mixed. Chose to keep it due to Tennessee law that strongly encourages naloxone prescribing.
Weight documentation of pediatric patients for nurses.	Do not fire when: Provider Primary Location = Vanderbilt Wilson County Adult Hospital	Discussed with clinicians, incorrect suggestions, need education. “If there was a significantly lower acceptance rate at any of the hospitals, it would make me think maybe staff education could need reinforcement if in fact the patients didn’t have a weight documented within a certain timeframe like at least 2-4 hours into their ED admission/visit.”
Your patient screened positive with symptoms concerning suicidal ideation based on the Columbia Suicide Severity Rating Scale. Please immediately assess patient further for safety and care recommendations and order appropriate Observation Precautions. [Critical]	Do not fire when: Provider Primary Department = BEHAVIORAL HEALTH CONSULT VPH	Discussed with clinicians, incorrect suggestions. “The provider’s chosen logon department is not relevant to the appropriateness of this safety BPA. The pattern of not accepting this BPA may provide us an opportunity for focused provider re-education.”

If all helpful suggestions were implemented, then for each BPA, the average decrease in alert firings would be 12.3% and the average relative change in acceptance rates would be 16.9%. Table 4 shows the number of firings and acceptance rates before and after applying the helpful generated suggestions grouped by BPAs.

**Table 4.** Comparisons of original and predicted firing counts with acceptance rates (ARs) before and after using “helpful suggestions.”

BPA	Firings (original)	AR (original)	Firings (predicted)	AR (predicted)	Potential firings eliminated (%)	Relative increase in AR
This patient is In Transit and is not yet marked as In Bed. Click “Place in assigned bed” to confirm that the patient is in the bed.	398 556	0.25	259 964	0.38	138 592 (34.8%)	52%
Admission medication reconciliation is incomplete, there are home medications that need a decision.	625 525	0.02	508 006	0.02	117 519 (18.8%)	25%
This patient is at risk for unintentional opioid overdose due to the following and a naloxone prescription is required to be offered by TN law.	46 506	0.13	37 508	0.17	8998 (19.3%)	31%
Hepatitis C diagnosis without Hepatitis B vaccine or immunity documented.	64 191	0.05	56 850	0.06	7341 (11.4%)	20%
Patient has not received a VTE risk score since admission—please choose from the following.	118 810	0.14	117 367	0.14	1443 (1.2%)	1%
This patient has one or more Shared Plan of Care FYI flags which may require your attention.	99 143	0.07	98 326	0.07	817 (0.8%)	1%
Patient has answered “Yes” to experiencing respiratory symptoms and fever. Give the patient a surgical mask to wear at all times.	12 940	0.12	12 255	0.13	685 (5.3%)	8%
This patient’s dosing weight is potentially out of date, please update the dosing weight.	5480	0.13	4953	0.14	527 (9.6%)	8%
Your patient does not meet criteria for RBC transfusion based on best-practice evidence.	2195	0.14	1724	0.18	471 (21.5%)	29%
Contraindicated—NSAIDs and Pregnancy	2196	0.10	1734	0.13	462 (21%)	30%
Warfarin Dosing Advisor	1422	0.32	1047	0.43	375 (26.4%)	34%
PATIENT WITH PNEUMONIA: Use the Community-Acquired Pneumonia (CAP) Antibiotic Advisor unless excluded.	1246	0.14	919	0.19	327 (26.2%)	36%
Premature infants born <32 weeks or <1500 g are at high risk for Retinopathy of Prematurity (ROP). Please confirm inclusion on the ROP exam list or indicate your reasons for opting out.	13 646	0.61	13 361	0.62	285 (2.1%)	2%
This patient is due for the flu vaccine. Please order or specify why the vaccine cannot be ordered.	18 115	0.52	17 834	0.53	281 (1.6%)	2%
This patient does not currently have a dosing weight.	5334	0.35	5067	0.37	267 (5%)	6%
(For NICU Attending) Premature infants born <31 weeks or <1500 gms are at high risk for Retinopathy of Prematurity. Please confirm inclusion on the ROP exam list or indicate your reasons for opting out.	10 703	0.60	10 513	0.62	190 (1.8%)	3%
Potentially high vancomycin level.	961	0.06	828	0.07	133 (13.8%)	17%
Patient may require VTE prophylaxis—open the panel below for VTE prophylaxis options or select an exclusion reason.	124 797	0.12	124 703	0.12	94 (0.1%)	0%

The evaluation dataset contained 524 970 firings for 112 BPAs with an overall acceptance rate of 12.2%. Of the 1727 suggestions generated, 702 corresponded to situations where no alerts occurred. Specifically, 425 suggestions related to retired BPAs and the other 277 suggestions were likely to have been included in the modifications.

## Discussion

In this study, we developed and evaluated a data-driven process to generate suggestions for improving alert criteria using XAI approaches. This approach could eliminate alert firings by 9.3% after implementing the suggestions validated by CDS experts. This approach can significantly reduce, but not eliminate, human intervention, and it generates fully transparent rules in a timely manner from user interactions with alerts while potentially being more accurate compared to ordinary rule-based models.

The effectiveness of the XAI approach in eliminating alert firings might be underestimated due to the robust manual alert review process at VUMC. From March to September in 2020, VUMC performed an intensive 6-month initiative to refine alert criteria, working with 28 clinicians.^34^ In addition to this, VUMC has monthly CDS governance meetings to review alerts with low acceptance rates. These regular and comprehensive manual review processes provide a valuable opportunity to compare the data-driven generated suggestions with those generated by manual review. However, as a result, some of the suggestions generated from the 2019 alert data had already been identified in subsequent manual reviews. Conversely, the XAI approach may eliminate a larger number of alert firings from institutions with limited resources to conduct manual reviews.

We presented an effective approach to identify improvements in alert criteria which a human might not be able to identify or identify in a timely manner. Through a comparative analysis of modification timelines, we found that only 62% of the helpful suggestions were implemented by 2021. Furthermore, 16% of these helpful suggestions were implemented after 2022, and 22% of helpful suggestions were not identified by human review. Several reasons led to these results. When experts reviewed alerts, it was difficult to consider hundreds of possible improvements. Reviewers might not consider clinical scenarios which are rare or outside their area of practice (such as patients in the OR, patients receiving an imaging procedure, patients on hospice). On the other hand, this data-driven process could learn from millions of user interactions with alerts to cover a much wider range of scenarios. From this perspective, one person manually reviewing an alert could provide the opinion of one healthcare provider, but the XAI-based process could generate suggestions that are more comprehensive for all providers at the institution.

More importantly, our research not only has benefits within the scope of CDS, but it also aims to improve the clinical process. For example, for the alert regarding weight documentation of pediatric patients, one generated suggestion was “Do not fire when: Provider Primary Location = Vanderbilt Wilson County Adult Hospital.” This suggestion was noted by a stakeholder for its importance to further explore the reason of low acceptance in this location and the potential to reinforce education on its use. It demonstrates that alert log data provides not only user acceptance of alerts, but also an opportunity to track practices and associated implementation process. Overall, this data-driven process transforms CDS alert knowledge maintenance into a learning health system by effectively utilizing user interaction data in the clinical setting. This would enable the CDS team to learn from experience and inform clinical improvements, leading to continuous enhancements in healthcare quality and efficiency.

Healthcare organizations and EHR vendors should consider developing or adopting automated methods to identify potential improvements to CDS.^35^ Right now, implementing the suggestions requires manual work to update CDS logic, but EHR vendors could also add tools to allow users to accept suggestions directly and automatically adjust the CDS logic accordingly. These automated methods complement other approaches to CDS improvement such as the Clickbusters process, review of user feedback, and monitoring.^34^^,^^36^ Additionally, the Epic EHR system includes an automatic tool “Tune-up” that suggests updates to minimize disruptions, focusing on features like popup and acknowledgment lockout periods, alert triggers, and provider-specific details such as type, department, and specialty. However, “Tune-up” is constrained to suggest modifications for only one feature at a time. The XAI approach could consider combinations of different features and generate suggestions with multiple features.

### Limitations

This study has several limitations. First, we developed and evaluated the data-driven process to generate suggestions using datasets from a single medical center. Exploring the capability of this data-driven process in other healthcare systems might add more value. Second, as a retrospective study, the impact of generated suggestions on patient outcomes and physician behaviors remains unknown.

### Future work

Future work in this area includes designing an interface for CDS experts to visualize the XAI process and evaluate model-generated suggestions. A real-time and user-friendly interface could facilitate the process of improving CDS alert criteria, as described above. Another direction is to conduct a multi-site prospective study to implement suggestions and evaluate changes in clinician behavior and clinical outcomes.

## Conclusion

In summary, we developed a data-driven process to generate suggestions for improving alert criteria using XAI techniques. Our approach could identify improvements to CDS that might be overlooked or delayed in manual reviews. Our study also unveils a secondary purpose for the XAI: to improve quality by discovering scenarios where CDS alerts are not accepted due to workflow, education, or staffing issues. It is important for healthcare organizations and EHR vendors to integrate such automated techniques to improve CDS tools.

## Supplementary Material

ocae019_Supplementary_Data

## Data Availability

The data underlying this article cannot be shared publicly due to patient healthcare data privacy protection requirements.
