# Supplementary material for: Leveraging explainable artificial intelligence to optimize clinical decision support
Source: J Am Med Inform Assoc. 2024 Feb 22;31(4):968–74. doi: 10.1093/jamia/ocae019 (PMC10990514; doi:10.1093/jamia/ocae019)
Supplement: ocae019_Supplementary_Data [file ocae019_supplementary_data.docx]

Method of Data Selection used in Anchor

Notably, because the locally interpretable method (Anchor) generated specific rules for each data instance, a limitation of this technique involved considerable computing time, particularly when handling large datasets. To address this problem, we applied a dynamic cluster analysis for each alert using k-means on the original predictive features. To determine the optimal number of clusters, we utilized the kneedle algorithm to identify the inflection point of the inertia.[1] Subsequently, we randomly selected 10 data points from each cluster to generate rules using the Anchor method.

**References**

1 Satopää V, Albrecht J, Irwin D, *et al.* Finding a ‘kneedle’ in a haystack: Detecting knee points in system behavior. In: *Proceedings - International Conference on Distributed Computing Systems*. 2011. 166–71. doi:10.1109/ICDCSW.2011.20

Table 1. The pros and cons of each metric.

| Odds Ratio | It assesses the likelihood of alert rejection within the suggested scenarios versus outside them. However, it does not consider the absolute numbers of acceptances and rejections. |
| --- | --- |
| Probability of Acceptance | It estimates the acceptance likelihood based on a Beta distribution, informed by acceptance and rejection counts within the suggestion. Its limitation is the exclusion of acceptance rates outside the suggestion's scope. |
| Decrease Rate | It evaluates the potential reduction of alert firings if the suggestion is applied. But it does not account for the acceptance-to-rejection ratio within the suggested context. |
| Confidence | It measures the proportion of rejections within the suggestion, yet does not consider the acceptance-to-rejection ratio in a broader context. |
| Interest | It reflects the co-occurrence of non-acceptance with the suggestions compared to a baseline, but it does not account for the number of non-acceptances both within and outside the suggestion. |
| Conviction | It compares the acceptance rates within the suggestion to a baseline, but overlooks the non-acceptance firings within the suggested scenario. |
| P-value of χ^2 | It evaluates the statistical relevance of non-acceptance to the suggestion, but it does not factor in the count of non-acceptances within and outside the suggestion. |

Table 2. Features used in the model.

| **Feature Category** | **Number of Features** | **Examples** |
| --- | --- | --- |
| Patient feature | 15 | Patient Sex, Patient Age, Patient Ethnic Group, Patient Department, medications per day, medications per month, office visits per year, hospital encounters per year, emergency visits per year, alcohol usage, race, office visits per month, hospital encounters per month, emergency visits per month, smoking status |
| Provider feature | 6 | Provider Type, Provider Primary Department, Provider Primary Location, Provider Primary Service Area, Provider Specialty, treatment team affiliation |
| Alert feature | 3 | BPA, Importance Level, BPA Trigger Action |
| Medication | 834 | (Number of medications, grouped by pharmclass)  1ST GEN ANTIHIST-DECON-ANALGESIC SALICYLATE, …, ZINC REPLACEMENT |
| Diagnosis | 279 | (Number of diseases, grouped by CCS categories)  diagnosis_1, …, diagnosis_279 |
| Alert-related orders and status | 233 | ALBUTEROL SULFATE 2.5 MG/3 ML (0.083 %) SOLUTION FOR NEBULIZATION [250]Canceled, WARFARIN 7.5 MG TABLET [8752]Sent |
| Count of problems | 1 | Count of problems |
| Lab | 74 | (Select labs with missing rate <80%)  HEMATOCRIT244, …, LACTIC ACID LEVEL4835 |
| Vitals | 12 | BMI (Calculated), BP_L, BP_H, Fall Risk Calculated Score, Heart Rate, Height, MAP (mmHg), Mental Status/RASS Score, Pulse, Resp, SpO2, Weight |
| Encounter | 4 | Encounter type, New provider encounter indicator, New specialty encounter indicator, New department encounter indicator |
| Chief complaint | 1231 | Flu symptoms, new patient, return, ... |

Table 3. Generated suggestions that were already changed, partial changed, or discussed with stakeholders.

| **Generated Suggestion** |  | **Change Date** | **Comment** |
| --- | --- | --- | --- |
| BPA: VUMC ED TRANSIT BIN - PATIENT NOT MARKED IN ROOM | | | |
| Provider Type = Resident Physician | already changed | 22-04-21 | same effect, the current BPA limits to Provider Type: Paramedic, registered nurse; technician |
| Provider Specialty = RESIDENT | already changed | 20-04-02 | same effect, the current BPA limits to Specialty: Emergency Medicine |
| Provider Specialty = PHARMACY | already changed | 20-04-02 | same effect, the current BPA limits to Specialty: Emergency Medicine |
| Provider Type = Pharmacist | already changed | 22-04-21 | same effect, the current BPA limits to Provider Type: Paramedic, registered nurse; technician |
| Provider Specialty = PEDIATRIC EMERGENCY MEDICINE | already changed | 20-04-02 | same effect, the current BPA limits to Specialty: Emergency Medicine |
| Provider Specialty = FELLOW | already changed | 20-04-02 | same effect, the current BPA limits to Specialty: Emergency Medicine |
| Provider Type = Research Coordinator - Non Clinical | already changed | 22-04-21 | same effect, the current BPA limits to Provider Type: Paramedic, registered nurse; technician |
| Provider Type = Social Worker | already changed | 22-04-21 | same effect, the current BPA limits to Provider Type: Paramedic, registered nurse; technician |
| Provider Specialty = GENERAL PEDIATRICS | already changed | 20-04-02 | same effect, the current BPA limits to Specialty: Emergency Medicine |
| Provider Primary Location_missing >= 0.50 | already changed | 20-04-02 | same effect, the rule suggests to exclude missing location, the current BPA limits to VUMC IP HOSPITAL LOCATIONS (VUH/VCH/VPH) |
| Provider Type = Medical Student | already changed | 22-04-21 | same effect, the current BPA limits to Provider Type: Paramedic, registered nurse; technician |
| Provider Specialty = PHYSICIAN ASSISTANT | already changed | 20-04-02 | same effect, the current BPA limits to Specialty: Emergency Medicine |
| Provider Type = Physician Assistant | already changed | 22-04-21 | same effect, the current BPA limits to Provider Type: Paramedic, registered nurse; technician |
| Provider Specialty = MEDICAL STUDENT | already changed | 20-04-02 | same effect, the current BPA limits to Specialty: Emergency Medicine |
| Provider Primary Department = PEDS PRIMARY CARE DOT 8 N [101039192] | already changed | 20-04-02 | same effect, the current BPA limits to VUMC IP HOSPITAL LOCATIONS (VUH/VCH/VPH) |
| Provider Primary Location = VUMC Adult One Hundred Oaks [101087] | already changed | 20-04-02 | same effect, the current BPA limits to VUMC IP HOSPITAL LOCATIONS (VUH/VCH/VPH) |
| Provider Type = Respiratory Therapist | already changed | 22-04-21 | same effect, the current BPA limits to Provider Type: Paramedic, registered nurse; technician |
| Provider Specialty = RESPIRATORY THERAPY | already changed | 20-04-02 | same effect, the current BPA limits to Specialty: Emergency Medicine |
| Provider Type = Fellow | already changed | 22-04-21 | same effect, the current BPA limits to Provider Type: Paramedic, registered nurse; technician |
| Provider Specialty = SOCIAL WORK | already changed | 20-04-02 | same effect, the current BPA limits to Specialty: Emergency Medicine |
| Provider Primary Department = PEDS PRIMARY CARE DOT 8 S [101039193] | already changed | 20-04-02 | same effect, the current BPA limits to Specialty: Emergency Medicine |
| Provider Primary Department = PEDS AFTER HOURS HENDERSONVILLE HVLNS [101046105] | already changed | 20-04-02 | same effect, the current BPA limits to VUMC IP HOSPITAL LOCATIONS (VUH/VCH/VPH) |
| Provider Primary Location = VUMC Hendersonville - Anderson [107164] | already changed | 20-04-02 | same effect, the current BPA limits to VUMC IP HOSPITAL LOCATIONS (VUH/VCH/VPH) |
| Provider Primary Department = ORTHOPAEDICS SPORTS MEDICINE HENDERSONVILLE [107164110] | already changed | 20-04-02 | same effect, the current BPA limits to VUMC IP HOSPITAL LOCATIONS (VUH/VCH/VPH) |
| Provider Specialty = ADULT-GERONTOLOGY ACUTE CARE NURSE PRACTITIONER | already changed | 20-04-02 | same effect, the current BPA limits to Specialty: Emergency Medicine |
| Provider Specialty = HOSPITALIST | already changed | 20-04-02 | same effect, the current BPA limits to Specialty: Emergency Medicine |
| Provider Primary Location = VUMC Adult Medical Center East [101014] | already changed | 20-04-02 | same effect, the current BPA limits to VUMC IP HOSPITAL LOCATIONS (VUH/VCH/VPH) |
| Provider Specialty = INTERNAL MEDICINE | already changed | 20-04-02 | same effect, the current BPA limits to Specialty: Emergency Medicine |
| Provider Type = Mental Health Specialist | already changed | 22-04-21 | same effect, the current BPA limits to Provider Type: Paramedic, registered nurse; technician |
| Provider Primary Location = VUMC VPH Psych Adult [107130] | already changed | 20-04-02 | same effect, the current BPA limits to VUMC IP HOSPITAL LOCATIONS (VUH/VCH/VPH) |
| Provider Specialty = CHILD LIFE | already changed | 20-04-02 | same effect, the current BPA limits to Specialty: Emergency Medicine |
| Provider Type = Pharmacy Technician | already changed | 22-04-21 | same effect, the current BPA limits to Provider Type: Paramedic, registered nurse; technician |
| Provider Primary Department = INTERNAL MEDICINE POD B OHO NORTH [101087207] | already changed | 20-04-02 | same effect, the current BPA limits to VUMC IP HOSPITAL LOCATIONS (VUH/VCH/VPH) |
| Provider Primary Department = PEDS PRIMARY CARE EVENING DOT 8 [101039217] | already changed | 20-04-02 | same effect, the current BPA limits to VUMC IP HOSPITAL LOCATIONS (VUH/VCH/VPH) |
| Provider Primary Location = VUMC Adult Village at Vanderbilt [101086] | already changed | 20-04-02 | same effect, the current BPA limits to VUMC IP HOSPITAL LOCATIONS (VUH/VCH/VPH) |
| Provider Primary Department = PHARMACOLOGY TVC 2 [101019121] | already changed | 20-04-02 | same effect, the current BPA limits to VUMC IP HOSPITAL LOCATIONS (VUH/VCH/VPH) |
| Provider Primary Department = BEHAVIORAL HEALTH THERAPY VPH [107130100] | already changed | 20-04-02 | same effect, the current BPA limits to VUMC IP HOSPITAL LOCATIONS (VUH/VCH/VPH) |
| Provider Specialty = OPHTHALMOLOGY | already changed | 20-04-02 | same effect, the current BPA limits to Specialty: Emergency Medicine |
| Provider Primary Department = PEDS GASTROENTEROLOGY DOT 10 [101039102] | already changed | 20-04-02 | same effect, the current BPA limits to VUMC IP HOSPITAL LOCATIONS (VUH/VCH/VPH) |
| Provider Specialty = NURSE PRACTITIONER ADULT GERONTOLOGY PRIMARY CARE | already changed | 20-04-02 | same effect, the current BPA limits to Specialty: Emergency Medicine |
| Provider Primary Department = INTERNAL MEDICINE HMG VAV [101086120] | already changed | 20-04-02 | same effect, the current BPA limits to VUMC IP HOSPITAL LOCATIONS (VUH/VCH/VPH) |
| Provider Primary Department = PULMONARY OHO NORTH [101087284] | already changed | 20-04-02 | same effect, the current BPA limits to VUMC IP HOSPITAL LOCATIONS (VUH/VCH/VPH) |
| Provider Type = Pharmacist Student | already changed | 22-04-21 | same effect, the current BPA limits to Provider Type: Paramedic, registered nurse; technician |
| Provider Primary Department = WALK IN CLINIC MELROSE [107140100] | already changed | 20-04-02 | same effect, the current BPA limits to VUMC IP HOSPITAL LOCATIONS (VUH/VCH/VPH) |
| Provider Primary Department = BEHAVIORAL HEALTH CONSULT VPH [107130102] | already changed | 20-04-02 | same effect, the current BPA limits to VUMC IP HOSPITAL LOCATIONS (VUH/VCH/VPH) |
| Provider Specialty = *Unspecified Specialty | discuss -> incorrect | | Stakeholder: "Unknown provider specialty is typically a nurse practitioner or resident. They still need to see this BPA since they do a lot of admissions." |
| Provider Type = Fellow | partial | 20-03-13 | partial effect, the current BPA limits to fellow with encounter type: Hospital Encounter |
| Provider Type = Pharmacist | partial | 20-03-13 | partial effect: the current BPA limits to pharmacist with encounter type: hospital encounter |
| Provider Specialty = PHARMACY | partial | 20-03-13 | partial effect, the current BPA limits to physician assistant with encounter type: hospital encounter |
| Provider Type = Utilization Manager | already changed | 20-03-13 | already changed, the current BPA not shown to utilization manager |
| Provider Type = Respiratory Therapist | already changed | 20-03-13 | already changed, the current BPA not shown to Respiratory Therapist |
| Provider Specialty = RESPIRATORY THERAPY | already changed | 20-03-13 | already changed, the current BPA not shown to Respiratory Therapist |
| Provider Primary Department = PULMONARY TVC B [101019124] | already changed | 20-12-10 | already changed, the current BPA excludes Location: VUMC The Vanderbilt Clinic |
| Provider Primary Location = VUMC The Vanderbilt Clinic [101019] | already changed | 20-12-10 | already changed, the current BPA excludes Location: VUMC The Vanderbilt Clinic |
| Provider Type = Medical Student | already changed | 20-03-13 | already changed, the current BPA not shown to medical student |
| Provider Primary Department = SURGERY TRAUMA TVC 3 [101019148] | already changed | 20-12-10 | already changed, the current BPA excludes Location: VUMC The Vanderbilt Clinic |
| Provider Specialty = MEDICAL STUDENT | already changed | 20-03-13 | already changed, the current BPA not shown to medical student |
| Provider Primary Department = NEPHROLOGY TVC 2 [101019120] | already changed | 20-12-10 | already changed, the current BPA excludes Location: VUMC The Vanderbilt Clinic |
| Patient Department = VCH 4E NEWBORN NURSERY [101001102] | already changed | 20-02-21 | already changed, the current BPA excludes department VCH 4E NEWBORN NURSERY [101001102] |
| Provider Primary Department = GASTROENTEROLOGY TVC 1 [101019152] | already changed | 20-12-10 | already changed, the current BPA excludes Location: VUMC The Vanderbilt Clinic |
| Provider Primary Department = PEDS PRIMARY CARE EVENING DOT 8 [101039217] | already changed | 20-12-10 | already changed, the current BPA excludes Location: VUMC Doctors' Office Tower |
| Provider Type = Nurse Anesthetist | partial | 20-03-13 | partial effect, the current BPA limits to Nurse Anesthetist with encounter type: hospital encounter |
| BPA: VUMC AMB BASE NALOXONE CO-PRESCRIBING | | | |
| ENC_TYPE = Refill | discuss |  | This exclusion was discussed in CDS committee meetings several times. The CDS committee chose to keep refill encounters. |
| Provider Specialty = HOSPICE AND PALLIATIVE MEDICINE | discuss -> correct | 22-06-30 | Stakeholder: " I think it would make sense to exclude them, but I would have to check with our opioid executive committee and legal." The current BPA has added "Palliative care" as one of acknowledge reasons |
| REFILL REQUEST >= 0.50 | discuss |  | This exclusion was discussed in CDS committee meetings several times. The CDS committee chose to keep refill encounters. |
| Provider Specialty = OCCUPATIONAL MEDICINE | discuss |  | Stakeholder: "still want to show to them for education" |
| BPA: BASE HEPATITIS C AND NO HEPATITIS B IMMUNITY | | | |
| Patient Department_ZZZ-HEPATOLOGY TVC 1 [101019118] > 0.50 and Provider Type_Patient Access <= 0.50 and Provider Primary Department_TRANSPLANT SERVICES VAV 3 [101086113] <= 0.50 | partial | 22-09-28 | exclude provider type "patient access" on 9/28/22; retired on 04/03/23 |
| Patient Department_ZZZ-HEPATOLOGY TVC 1 [101019118] > 0.50 and Provider Type_Patient Access <= 0.50 and Provider Specialty_PHYSICIAN ASSISTANT <= 0.50 | partial | 22-09-28 | exclude provider type "patient access" on 9/28/22; retired on 04/03/23 |
| Patient Department_ZZZ-HEPATOLOGY TVC 1 [101019118] > 0.50 and Provider Type_Patient Access <= 0.50 and Provider Type_Physician Assistant <= 0.50 | partial | 22-09-28 | exclude provider type "patient access" on 9/28/22; retired on 04/03/23 |
| Provider Type = Pharmacy Technician | already changed | 22-09-28 | exclude provider type "Pharmacy Technician" on 9/28/22; retired on 04/03/23 |
| ENC_TYPE = Specialty Pharmacy Services | already changed | 19-11-07 | earlier than 11/7/19 and after 1/19/19 limit the encounter types to "office visit, telemedicine, appointment, follow-up, orders only" |
| BPA: VUMC IP BASE SHARED PLANS OF CARE FYI FLAG ALERT | | | |
| ENC_TYPE = Documentation | already changed | 23-03-16 | already changed, exclude enc type =documentation |
| BPA: VUMC BASE RX UPDATE DOSING WEIGHT VCH | | | |
| Patient Department = VCH 10A PCICU [101001212] | discuss -> incorrect | | Stakeholder: "This is correct, the BPA should only show in the NICUs, PICU, and PCICU." |
| Provider Type_Pharmacist >= 0.50 | discuss -> correct | | Stakeholder: "They already saw weight, could turn off" |
| BPA: VUMC BASE ROP FYI FLAG FOR PREMATURE INFANTS | | | |
| RETURN = TRUE | discuss -> correct | | Stakeholder: "It could limit to inpatient only." |
| NEW PATIENT = TRUE | discuss -> correct | | Stakeholder: "It could limit to inpatient only." |
| BPA: VUMC BASE ROP FYI FLAG FOR PREMATURE INFANTS (FOR NICU ATTENDING) | | | |
| RETURN = TRUE | discuss -> correct | | Retired. Stakeholder: "It could limit to inpatient only." |
| BPA: VUMC IP BASE ADULT PROVIDER FLU IMMUNIZATION EXIT CHECK | | | |
| Patient Department = VPH ADULT PARTIAL HOSPITALIZATION [101002107] | already changed | 20-12-17 | already changed, added an exclusion: department VPH ADULT PARTIAL HOSPITALIZATION [101002107] |
| ENC_TYPE = Office Visit | discuss -> correct | | Stakeholder: "There would be no harm in adding exclusions of Office Visit and Orders Only." |
| ENC_TYPE = Orders Only | discuss -> correct | | Stakeholder: "There would be no harm in adding exclusions of Office Visit and Orders Only." |
| BPA: VUMC IP BASE VTE PROPHYLAXIS PADUA MEDICAL - NO SCORE OR EXCLUSION REASON AT 4 HOURS | | | |
| Provider Primary Department_CARDIOLOGY EP MCE 5 [101014101] >= 0.50 | partial |  | Retired. Partial changed, limited to VUMC IP HOSPITAL LOCATIONS, this department is not in this location. |
| BPA: VUMC BASE RX NO DOSING WEIGHT VCH | | | |
| Provider Specialty = PHARMACY | discuss -> correct | | Stakeholder: "They already saw weight. It could turn off." |
| BPA: COLUMBIA SUICIDE RISK - PAS PROVIDER | | | |
| Provider Primary Department_BEHAVIORAL HEALTH CONSULT VPH [107130102] >= 0.50 | discuss -> incorrect | | Stakeholder: "This is a BPA where 'accepting' or 'not accepting' is more about provider temperament than anything about the BPA. It is not helpful for the provider department." |
| Provider Primary Department = BEHAVIORAL HEALTH CD VPH [107130104] | discuss -> incorrect | | Stakeholder: "This is a BPA where 'accepting' or 'not accepting' is more about provider temperament than anything about the BPA. It is not helpful for the provider department." |
| BPA: VUMC ED PATIENT - ORDER HOME INSULIN | | | |
| Provider Primary Department = VUMC EVENT ASSESSMENT [101000394] | discuss -> incorrect | | Stakeholder: "I don’t know what department this is. I would imagine that ED providers might also be associated with Event Assessment." |
| BPA: COMMUNICABLE DISEASE SYMPTOMS | | | |
| Provider Type = Technician | discuss |  | retired |
| BPA: AMB VUMC PREDICT DGI2 (WARFARIN) | | | |
| CCS_Menstrual disorders <= 2.00 and Patient Department_CARDIOLOGY COAG GREEN HILLS VIR [101095102] > 0.50 and ENC_TYPE_Communication <= 0.50 | discuss -> correct | | Stakeholder: "Yes, make sense. almost none of those patients are new to warfarin therapy and so the coag clinic is going to use INR history for most patients. We potentially could do a combination of department and some kind of different lookback than we are doing now – they’ll occasionally need to adjust a patient who comes in on standard therapy. I’m pretty sure they are clicking “this is not new therapy” which is the initial warfarin screen" |
| ENC_TYPE = Anticoagulation Visit | discuss -> correct | | Stakeholder: "Yes, make sense. almost none of those patients are new to warfarin therapy and so the coag clinic is going to use INR history for most patients. We potentially could do a combination of department and some kind of different lookback than we are doing now – they’ll occasionally need to adjust a patient who comes in on standard therapy. I’m pretty sure they are clicking “this is not new therapy” which is the initial warfarin screen" |
| CCS_Menstrual disorders <= 1.50 and Patient Department_CARDIOLOGY COAG GREEN HILLS VIR [101095102] > 0.50 and ENC_TYPE_Communication <= 0.50 | discuss -> correct | | Stakeholder: "Yes, make sense. almost none of those patients are new to warfarin therapy and so the coag clinic is going to use INR history for most patients. We potentially could do a combination of department and some kind of different lookback than we are doing now – they’ll occasionally need to adjust a patient who comes in on standard therapy. I’m pretty sure they are clicking “this is not new therapy” which is the initial warfarin screen" |
| BPA: ED PEDS WEIGHT DOCUMENTED - NURSE | | | |
| Provider Primary Location = Vanderbilt Wilson County Adult Hospital [101341] | discuss -> incorrect | | Stakeholder: "If there was a significantly lower acceptance rate at any of the hospitals, it would make me think maybe staff education could need reinforcement if in fact the patients didn’t have a weight documented within a certain timeframe like at least 2-4 hours into their ED admission/visit. There would be harm in restricting this BPA since documenting a weight in the pediatric population is so vital to their management of care." |
| BPA: RED BLOOD CELL GUIDANCE FOR ORDERING | | | |
| BPA Trigger Action_Sign Treatment Plan Order >= 0.50 | discuss -> correct | | Stakeholder: "The treatment plans in heme/onc also have chronic transfusions in them." |
| BPA Trigger Action_Release Treatment Plan Order >= 0.50 | discuss -> correct | | Stakeholder: "The treatment plans in heme/onc also have chronic transfusions in them." |
| Patient Department = ONCOLOGY INFUSION TVC 2 [101019108] | discuss -> correct | | Stakeholder: “Those are our same heme/onc patients going to the infusion center for their transfusion." |
| Provider Specialty = HEMATOLOGY/ONCOLOGY | discuss |  | Stakeholder: "Consider excluding patients with Sickle cell anemia, Beta thalassemia major, Diamond Blackfan anemia, and Fanconi’s anemia" |
| BPA: VUMC IP BASE CAP DIAGNOSIS AND NLP POSITIVE FOR SMARTFORM - ICE-CAP STUDY | | | |
| BPA Trigger Action_Open Orders Sidebar <= 0.50 | Partial | 2/7/20 | retired. This rule means to exclude other BPA trigger action except the open orders sidebar.  The BPA removed "General BPA section" and "Dispo Workspace" and "Open Patient Chart" BPA trigger actions. Potential trigger actions are Open Order Entry activity and Open Orders Sidebar. |
| BPA Trigger Action_Open Orders Sidebar <= 0.50 and Asian_True <= 0.50 and ROSACEA AGENTS, TOPICAL <= 0.50 | Partial | 2/7/20 | retired. This rule means to exclude other BPA trigger action except the open orders sidebar.  The BPA removed "General BPA section" and "Dispo Workspace" and "Open Patient Chart" BPA trigger actions. Potential trigger actions are Open Order Entry activity and Open Orders Sidebar. |
| BPA Trigger Action = Dispo Workspace | already changed | 2/7/20 | retired. Already changed. |
| BPA Trigger Action_Open Orders Sidebar <= 0.50 and Height > 27.04 and CCS_Asthma <= 3.50 | Partial | 2/7/20 | retired. This rule means to exclude other BPA trigger action except the open orders sidebar.  The BPA removed "General BPA section" and "Dispo Workspace" and "Open Patient Chart" BPA trigger actions. Potential trigger actions are Open Order Entry activity and Open Orders Sidebar. |
| BPA Trigger Action_Open Orders Sidebar <= 0.50 and COUGH AND/OR COLD PREPARATIONS <= 0.50 and CCS_Asthma <= 3.50 | Partial | 2/7/20 | retired. This rule means to exclude other BPA trigger action except the open orders sidebar.  The BPA removed "General BPA section" and "Dispo Workspace" and "Open Patient Chart" BPA trigger actions. Potential trigger actions are Open Order Entry activity and Open Orders Sidebar. |
| BPA Trigger Action_Open Orders Sidebar <= 0.50 and BP_H <= 98.50 | Partial | 2/7/20 | retired. This rule means to exclude other BPA trigger action except the open orders sidebar.  The BPA removed "General BPA section" and "Dispo Workspace" and "Open Patient Chart" BPA trigger actions. Potential trigger actions are Open Order Entry activity and Open Orders Sidebar. |
| BPA: VUMC IP BASE RT BRONCHODILATOR PROTOCOL | | | |
| Provider Primary Location = VUMC LEBANON WEST MAIN STREET [107139] | discuss -> incorrect | | Stakeholder: "I wouldn’t put a lot of weight on the 'provider primary location' since our providers practice at multiple locations. This BPA is already limited to only show at VUAH." |
| BPA: BASE VWCH MED HISTORY PHARM COMPLETE | | | |
| Provider Primary Location_VUMC LEBANON WEST MAIN STREET [107139] >= 0.50 | discuss -> incorrect | | Stakeholder: "I wouldn’t put a lot of weight on the “provider primary location” since our providers practice at multiple locations. This BPA is already limited to only show at VWCH." |
| BPA: BASE VUMC IP FAMOTIDINE IV TO PO BPA | | | |
| Patient Department = ZZZ-VUH 4RW GENERAL SURGERY [101000111] | discuss -> incorrect | | retired. Stakeholder: "I would not exclude 4RW because those patients tend to be the lower acuity patients who would likely be good candidates for switching to PO." |
| BPA: VUMC RX NSAID/PREGNANCY | | | |
| Patient Department_VUH 4E POST PARTUM [101000109] >= 0.50 | already changed | 8/6/20 | Already changed, add an exclusion criteria: exclude Dep = VUH 4E POST PARTUM [101000109] |
| BPA: VUMC IP BASE VTE PROPHYLAXIS PADUA MEDICAL - SCORE / EXCLUSION EXISTS BUT NOT ON PROPHYLAXIS | | | |
| ABNORMAL BLEEDING = TRUE | discuss |  | The BPA is: Patient may require VTE prophylaxis - open the panel below for VTE prophylaxis options or select an exclusion reason. Stakeholder: "We simplified the contraindication in 2021-11-11. It has Bleeding or high risk for bleeding as one of acknowledge reason." |
| BPA: BASE VUMC RX HIGH VANCOMYCIN LEVEL SIGN HAS CONSULT (VUH WVCH VBCH AND VTHH) | | | |
| LYMPHS1810218 <= 6.55 | discuss -> incorrect | | Stakeholder: "I think this makes sense and should be kept. If Lymphs are low on a patient, then they might want to be more aggressive with vancomycin therapy." |
